# Effective dosage of enoxaparin for intensive care patients

**DOI:** 10.1186/cc12287

**Published:** 2013-03-19

**Authors:** S Robinson, A Zincuk, U Larsen, P Toft

**Affiliations:** 1 Odense University Hospital, Odense, Denmark

## Introduction

We hypothesized that higher doses of enoxaparin would improve thromboprophylaxis without increasing the risk of bleeding. Critically ill patients are predisposed to venous thromboembolism, leading to increased risk of adverse outcome [1]. Peak anti-factor Xa (anti-Xa) levels of 0.1 to 0.4 IU/ml, 4 hours post administration of enoxaparin, reflect adequate thromboprophylaxis for medico-surgical patients.

## Methods

The sample population consisted of 78 patients, randomized to receive subcutaneous (s.c.) enoxaparin: 40 mg ×1 (control group), versus 30 mg ×2, 40 mg ×2 or 1 mg/kg ×1 (test groups) for a period of 3 days. Anti-Xa activity was measured at baseline, and at 4, 12, 16 and 24 hours post administration on each day. Patients did not differ significantly between groups.

## Results

**Figure 1:**
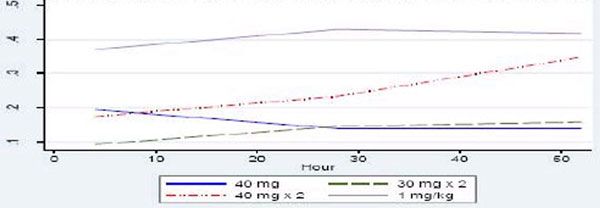
**Peak anti-Xa over 3 days with varying doses of enoxaparin**.

On day 1 of administration, doses of 40 mg ×1 and 40 mg ×2 yielded similar mean peak anti-Xa of 0.20 IU/ml and 0.17 IU/ml respectively, while a dose of 30 mg ×2 resulted in subtherapeutic levels of anti-Xa (0.08 IU/ml). Patients receiving 1 mg/kg enoxaparin achieved near-steady-state levels from day 1 with mean peak anti-Xa levels of 0.34 IU/ml. Steady-state anti-Xa was achieved for all doses of enoxaparin at day 3. At steady state, mean peak anti-Xa levels of 0.13 IU/ml and 0.15 IU/ml were achieved with doses of 40 mg ×1 and 30 mg ×2 respectively. This increased significantly to 0.33 IU/ml and 0.40 IU/ml for doses of 40 mg ×2 and 1 mg/kg enoxaparin respectively (*P *= 0.0000) (Figure 1). A dose of 1 mg/kg enoxaparin yielded therapeutic anti-Xa levels for over 80% of the study period. There were no adverse effects.

## Conclusion

Current standard s.c. doses of 40 mg ×1 enoxaparin (Europe) or 30 mg ×2 (North America) yield levels of anti-Xa thought to be subtherapeutic for critically ill patients. A weight-based dose yielded the best anti-Xa levels, allowed the establishment of near-steady-state levels from the first day of enoxaparin administration, and may thus be more appropriate, convenient, and effective. A new study using 1 mg/kg enoxaparin s.c. with clinical endpoints has recently been approved by the Danish Ministry of Health.
